# Ocular residual and corneal astigmatism in a clinical population of high school students

**DOI:** 10.1371/journal.pone.0194513

**Published:** 2018-04-09

**Authors:** Zhao Chen, Luoru Liu, Chenglin Pan, Xiaoning Li, Lun Pan, Weizhong Lan, Zhikuan Yang

**Affiliations:** 1 Aier Institute of Optometry and Vision Science, Aier School of Ophthalmology, Central South University, Changsha, Hunan Province, China; 2 Anyang Eye Hospital, Anyang, Henan Province, China; Wenzhou Medical University Eye Hospital, CHINA

## Abstract

**Purpose:**

Total refractive astigmatism is usually the first consideration that guides the selection of contact lens type (e.g., spherical or toric), while the ocular source of the astigmatism is a second, but more important consideration, for the final clinical decision. This study was conducted to provide detailed data on this topic by evaluating astigmatic components in Chinese adolescents.

**Methods:**

Participants were recruited from healthy high school students undergoing an annual ocular examination at a local hospital. Total astigmatism (TA), corneal astigmatism (CA), and ocular residual astigmatism (ORA) were determined by a Hartmann-Shack wavefront analyzer system (KR-1W, Topcon) with the natural pupil. The axis relationship between CA and ORA was placed into three categories: on-axis, defined as an axis with a difference of 0 ± 10°; opposite-axis, a difference of 90 ± 10°; and the rest defined as oblique-axis.

**Results:**

The study consisted of 1,466 students (57.84% girls, age: 16.49 ± 1.05 years). ORA was present in 83.97%, 66.64%, and 45.23% of participants, according to the various criteria for astigmatism (≥ 0.50 D, ≥ 0.75 D, and ≥ 1.00 D, respectively). While with-the-rule was the most common axis orientation for both TA (76.28%) and CA (89.94%), against-the-rule predominated in ORA (93.82%; χ^2^ = 1688.544, *p* < 0.001). Opposite-axis was the major type of axis difference (90.96%) of clinical significance (i.e., ≥ 1.00 D) between CA and ORA, which also prevailed in all levels of TA (range: 56.25–82.26%).

**Conclusions:**

ORA is common in high school students and usually demonstrates a compensation relationship with CA, which should be taken into consideration when determining the design of contact lenses to correct refractive error.

## Introduction

Astigmatism, which is defined as the difference in the refractive error between two principal meridians of the eyeball, is the most common type of refractive error [1–4]. The total extent of astigmatism usually refers to the astigmatism of the eye as a complete ocular system (total astigmatism, TA), which can be measured by objective or subjective refraction. In fact, TA is the combined outcome of corneal astigmatism (CA) and ocular residual astigmatism (ORA). Although CA is theoretically composed of astigmatisms from both the anterior and posterior surfaces of the cornea, generally CA only refers to an astigmatism of the anterior corneal surface [5]. This is partly because of difficulties in measuring the posterior corneal surface and, in fact, the posterior corneal curvature has a relatively small impact on the total corneal astigmatism because of relatively small changes in the refractive index between the cornea and the aqueous humor [6]. On the other hand, ORA is defined as an astigmatism of the posterior corneal surface, plus that of both surfaces of the crystalline lens and any retinal astigmatism. [7,8].

For young adolescents, frame-glasses and contact lenses are the most common approaches used to correct refractive error. For frame-glasses, there is no need to differentiate where the astigmatism comes from, as this approach relies only on the amount of TA (i.e., the cylindrical power of the lens should be equal to the amount of TA). However, for contact lenses, especially for hard contact lenses, it is necessary to consider the contributions of both ORA and CA. Specifically, when the ORA and CA are in approximately the same axis, the TA will be reduced after correction by spherical contact lenses. In contrast, in cases where there is a significant difference in the axis between these two components, a contact lens with a toric design is necessary. Otherwise, the TA will increase after correction, because the CA will be reduced and the neutralizing effect of the crystalline lens on the CA will be lost. For instance, for a patient with 0.25 D of TA that is induced by 1.00 D x 6 of CA and 1.25 D x 105 of ORA, a spherical hard contact lens would result in substantial residual astigmatism.

Although CA and ORA have been widely investigated in adults [9–11], much less is known about their effects in young adolescents. Given that both the amount and the axis of CA and ORA change with age [12–14], the findings known for adults may not necessarily apply to young adolescents. Thus, the purpose of this study was to investigate the CA and ORA in adolescent subjects, with the aim of providing important data for decisions regarding the best approach for correcting refractive error.

## Materials and methods

### Subjects

The study was approved by the Ethics Committee of Aier School of Ophthalmology, Central South University and adhered to the tenets of the Declaration of Helsinki. Informed written consent was obtained from at least one parent. The study population was recruited from high school students who underwent an ocular health examination at Anyang Hospital (Henan Province, China). This hospital was selected because it was designated by the local Ministry of Education as the exclusive medical unit for performing annual ocular examinations for the students of the whole city. More details about the hospital and the characteristics of the students examined at this hospital can be found in the literature about the Anyang Eye Study [15–17]. Subjects with corneal diseases, cataracts and crystal lens dislocations, or any surgical history involving the eyes were excluded from the study.

### Procedures

Different types of astigmatisms were determined by a Hartmann-Shack wavefront analyzer system (KR-1W, Topcon, Tokyo, Japan). Briefly, the device first produces corneal and ocular wavefront aberrations on the same axis through the embedded Hartmann-Shack wavefront system. After that, using the same reference for centration allows for an accurate calculation of ORA in a relatively short time [6,18,19]. Three repeated automatic consecutive measurements of astigmatism were taken by an experienced optometrist. The measurements were conducted with the right eye only, with the natural pupil, and under quiet, highly mesopic conditions (8 lux). Although the device could produce data from both 6-mm and 4-mm pupil sizes, only data from the 4-mm pupil sizes were utilized in the following report to ensure the availability of data for all participants. Readings were considered valid according to the guidelines of the manufacturer. In order to validate the methodology, prior to the hospital-based study, a pilot study was conducted to compare the results of astigmatisms before and after cycloplegia. The cycloplegia was induced by three drops of Mydrin-P (0.5% tropicamide + 0.5% phenylephrine hydrochloride; Santen Pharmaceuticals, Osaka, Japan), and measurements were performed 30 minutes after the third drop of Mydrin-P was administered. The cycloplegic effect of this protocol has been reported in our previous studies [20–22].

### Definitions

For descriptions of the distribution of the astigmatic axis, a with-the-rule (WTR) astigmatism was defined as having a cylinder axis of 180° ± 30°, and an against-the-rule (ATR) astigmatism as 90° ± 30°. Oblique astigmatism was defined as having cylinder axes from 31°–59° or 121°–149°. The cylinder power was expressed in negative form. Vector decomposition, modified from Thibos et al. [23], was employed to convert the refractive and corneal cylinders into Cartesian (J_0_) and oblique (J_45_) vectors using the following equations:

$$J_{0}=-C/2cos\left(2\alpha \right)$$

$$J_{45}=-C/2sin\left(2\alpha \right)$$

where C is the negative-cylinder power and the angle α is the cylinder axis. J_0_ is the Jackson cross-cylinder power at axis 0° and 90°, and J_45_ is the Jackson cross-cylinder power at axis 45° and 135°. A positive J_0_ corresponds to a WTR astigmatism, while a negative J_0_ corresponds to an ATR astigmatism. A positive J_45_ indicates a negative cyclinder with axis at 45° and the most positive power at 135°, while a negative J_45_ indicates a negative cyclinder with axis at 135° and the most positive power at 45°. For the analysis of the axis difference between the ORA and CA, subjects were classified into three categories: on-axis, opposite-axis, and oblique-axis. In agreement with a study by Eom et al. [6], on-axis was defined as an axis difference between the ORA and CA of 0 ± 10°, opposite-axis was defined as a difference of 90 ± 10°, and the rest of the cases were defined as oblique-axis.

In addition, different criteria for the extent of astigmatism were also used in the current study (cylinder powers ≥ 0.50 D, ≥ 0.75 D, and ≥ 1.00 D) to facilitate comparison with other studies.

To indicate whether ORA can reduce TA by counterbalancing CA, a compensation factor (CF) was adopted in the current study, defined as CF = 1 –TA / CA [24,25]. In this formula, TA and CA are absolute values. Obviously, when CF > 0, the CA is counterbalanced by the ORA; when CF = 0, no ORA is indicated; and when CF < 0, the ORA synergizes with the CA, leading to an increase in the TA.

### Statistical analysis

Statistical analyses were performed using the Statistical Package for the Social Sciences (SPSS Inc., V. 16.0; Chicago, Illinois, USA). Quantitative data were first tested for normality using the Kolmogorov-Smirnov method. If normality was confirmed, the data were expressed as the mean ± SD, unless the median was applied instead. To test the validation of non-cycloplegic measurement of astigmatism, paired t-test and intraclass correlation coefficient (ICC) were performed if the validation data were normal. Quantitative variables with normality were compared by using t-tests, while quantitative variables with non-normality were compared by the Mann-Whitney test. Categorized variables were compared using the χ^2^ test or Fisher's exact test, if sample size (N) <40 or any theoretical frequency (T)<1 or P value of χ2 test is close to α. Pairwise tests were further performed by adjusting the P values using Bonferroni if significant multiple χ^2^ test was found. The Pearson correlation was used to investigate the correlation of the vector power between the ORA and CA. Results were considered statistically significant when the two-tailed *p* < 0.05.

## Results

### Validation of non-cycloplegic measurement of astigmatism

Table 1 shows the results of astigmatism measurements taken by the KR-1W before and after cycloplegia in 25 volunteers (60% girls). They were aged between 15–18 years with a mean age of 16.48 ± 1.05 years. The paired t-test confirmed that there was no significant difference in either the J_0_ or J_45_ of astigmatism before and after cycloplegia, indicating that it was justifiable to measure the astigmatism in the large-scale population with only the natural pupil. And the Intraclass Correlation Coefficient analysis (ICC) also indicate good consistency.

**Table 1:** Astigmatism before and after cycloplegia.

	J0 Vector				J45 Vector			
	Pre-Cylco	Post-Cylco	P*	ICC(95%CI)	Pre-Cylco	Post-Cylco	P*	ICC(95%CI)
**TA**	0.31±0.35	0.34±0.34	0.116	0.952(0.895,0.979)	-0.14±0.17	-0.03±0.17	0.453	0.904(0.794,0.956)
**CA**	0.58±0.42	0.61±0.43	0.214	0.957(0.905,0.981)	-0.04±0.20	-0.05±0.20	0.656	0.948(0.886,0.977)
**ORA**	-0.29±0.29	-0.26±0.29	0.117	0.923(0.833,0.965)	0.00±0.15	0.01±0.14	0.279	0.894(0.775,0.952)

TA, total astigmatism; CA, corneal astigmatism; ORA, ocular residual astigmatism; J0 = Jackson cross cylinder power at axis 0 and 90 degrees; J45 = Jackson cross cylinder power at axis 45 and 135 degrees; ICC, Intraclass Correlation Coefficient.

* indicates the significance of the change from baseline according to paired *t*-test.

In comparing the demographic data between the validation study and the main study, no significant difference was found (Table 2), further confirming that the justification for measuring astigmatism with natural pupil in the valuation study could be applied to the main study.

**Table 2:** Demographic characteristics between the validation study and the main study.

Variables	validation study	main study	*P*
*n*	25	1466	
Gender, Male, n(%) ^a^	10(40.0)	618(42.2)	0.829
Age, mean±SD ^b^	16.48±1.05	16.49±1.05	0.945
Sph, median(min,max) ^c^	-2.86(-12.13,0.97)	-2.93(-10.77,5.90)	0.700

Sph,Spherical refractive error

a. Chi-Square test.

b. Student-t test.

c. Nonparametric test: Mann-Whitney test.

### Percentage of astigmatism

In the present study, 1,466 high school students (57.8% girls) were examined for an astigmatism. The mean ± SD of the age was 16.49 ± 1.05 years (range: 15–18 years). The percentage of TA, CA, and ORA in this population is shown in Table 3. The percentage of TA ≥ 0.50 D (50.1%) was considerably lower than that of CA (90.5%) or ORA (84.0%). This was also the case for the criteria of astigmatism ≥ 0.75 D and ≥ 1.00 D. There was no significant difference in the percentage of TA between boys and girls for all criteria (all *p* > 0.05). However, for CA and ORA, girls tended to show a higher percentage of astigmatism, despite the fact that not all comparisons reached a statistically significant level.

**Table 3:** Percentage of TA, CA, and ORA by different criteria and gender.

		≥0.50D			≥0.75D			≥1.00D		
		%	(95% CI)	P	%	(95% CI)	P	%	(95% CI)	P
TA										
All	1466	50.1	(47.5~52.6)		28.3	(26.0~30.6)		17.3	(15.3~19.2)	
Boys	618	50.8	(46.9~56.8)	Reference	27.0	(23.5~30.5)	Reference	16.7	(13.7~19.6)	Reference
Girls	848	49.5	(46.2~52.9)	0.628	29.3	(26.2~32.3)	0.351	17.7	(15.1~20.3)	0.609
CA										
All	1466	90.5	(89.0~92.0)		78.2	(76.1~80.4)		64.4	(61.9~66.8)	
Boys	618	88.5	(86.0~91.0)	Reference	76.1	(72.7~79.4)	Reference	61.5	(57.6~65.3)	Reference
Girls	848	92.0	(90.5~93.3)	0.025*	79.8	(77.1~82.5)	0.083	66.5	(63.3~69.7)	0.047*
ORA										
All	1466	84.0	(82.1~85.9)		66.6	(64.2~69.1)		45.2	(42.7~47.8)	
Boys	618	82.9	(79.9~85.8)	Reference	65.2	(61.5~69.0)	Reference	42.9	(39.0~46.8)	Reference
Girls	848	84.8	(82.4~87.2)	0.317	67.7	(64.5~70.8)	0.32	46.9	(43.6~50.3)	0.024*

* indicates statistically significance.

### Frequency distribution of the axis of astigmatism

Given that a clinically significant threshold of astigmatism is generally set to 1.00 D, the distribution of the axis of TA, CA, and ORA of astigmatism ≥ 1.00 D is presented in Table 4. It was noted that WTR (88.9%) was the most common type of TA, followed by ATR (7.1%) and then the oblique (4.0%). Similarly, WTR (99.4%) predominated in the axis of CA, followed by oblique (0.4%) and ATR (0.2%). In comparison, ATR (99.4%) was the most common in ORA, followed by oblique (0.3%) and then WTR (0.3%). There was a significant difference in the distribution of the axis among TA, CA, and ORA (χ^2^ = 2300, *p* < 0.001). However, the percentage of the axis orientations between the genders was not significant for all three types of astigmatism (Fisher's exact test: all *p* > 0.05).

**Table 4:** Percentage of different types of axes of astigmatism greater than or equally 1.00D.

		WTR		ATR		Oblique		
N		%	(95% CI)	%	(95% CI)	%	(95% CI)	P
**TA**								
**All**	253	88.9	(85.1~92.8)	7.1	(5.6~8.8)	4.0	(2.8~5.3)	
**Boys**	103	91.3	(89.4~93.0)	5.8	(4.4~7.4)	2.9	(1.9~4.1)	Reference
**Girls**	150	87.3	(82.0~92.7)	8.0	(6.4~9.8)	4.7	(3.4~6.1)	0.633
**CA**								
**All**	944	99.4	(98.9~99.9)	0.2	(0.0~0.6)	0.4	(0.1~0.9)	
**Boys**	380	99.5	(98.5~99.9)	0.3	(0.0~1.0)	0.3	(0.0~1.0)	Reference
**Girls**	564	99.3	(98.4~99.8)	0.2	(0.0~0.7)	0.5	(0.1~1.3)	1.000
**ORA**								
**All**	663	0.3	(0.0~0.9)	99.4	(98.7~95.5)	0.3	(0.0~0.9)	
**Boys**	265	0.0	0	99.6	(98.5~100.0)	0.4	(0.0~1.5)	Reference
**Girls**	398	0.5	(0.1~1.4)	99.3	(98.7~99.9)	0.3	(0.0~1.0)	0.769

Table 5 shows the proportion of each type of axis difference between ORA and CA. It was observed that the opposite-axis (58.4%) accounted for the major type of relationship for astigmatisms ≥ 1.00 D, followed by the oblique-axis (40.5%) and the on-axis (1.1%). This characteristic was more obvious in subjects in which the CA/ORA was more severe than 1.00 D: opposite-axis (91.0%), oblique-axis (9.0%), and on-axis (0.0%).

**Table 5:** The axis relationship between ocular residual and corneal astigmatism.

	On-Axis		Oblique-Axis		Opposite-Axis	
	n	(%)	n	(%)	n	(%)
**ocular residual and corneal astigmatism <1.00 D**	10	(1.1)	361	(40.5)	520	(58.4)
**ocular residual and corneal astigmatism ≥ 1.00 D**	0	(0.0)	52	(9.0)	523	(91.0)
**All**	10	(0.7)	413	(28.2)	1043	(71.2)

**Fig 1:**
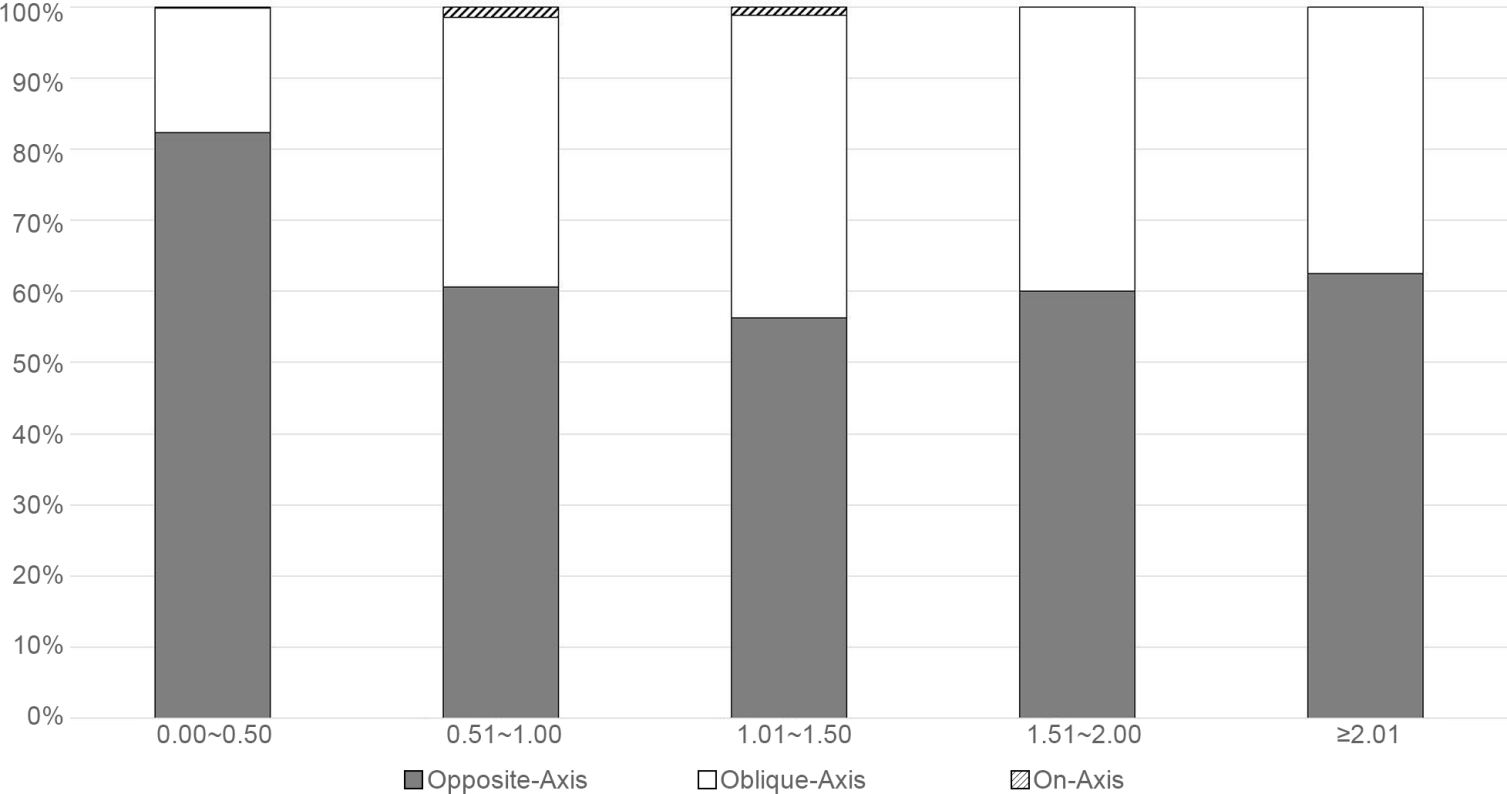
Proportion of the three types of axis relationships between CA and ORA in different levels of TA. On-axis: axis difference between the ORA and CA of 0 ± 10°, opposite-axis: axis difference of 90 ± 10°, with the remaining considered as oblique-axis.

When TA was taken into account and categorized into different magnitudes with intervals of 0.50 D, it was shown that the opposite-axis was still the major type of relationship between the ORA and CA for all levels of TA (Fig 1). The constituent ratio of the opposite-axis for each level of TA ranged from 56.3–82.3%. Although there was a significant difference in the proportion of the three types of axis relationships among different levels of TA (χ^2^ test, *p* < 0.001), there was no obvious trend for the change in proportion to the increased TA. In general, except for the group TA ≤ 0.50 D in which the opposite-axis accounted for 82.3%, this percentage was approximately 60% in all of the other groups.

Table 6 shows the CF percentage for all of the participants in the study, suggesting that the CA and ORA showed a counterbalancing relationship in the majority of subjects.

**Table 6:** Compensation factor for the relationship between ocular residual and corneal astigmatism.

	n	*%*
**CF>0**	1314	89.6
**CF = 0**	4	0.3
**CF<0**	148	10.1

**Fig 2:**
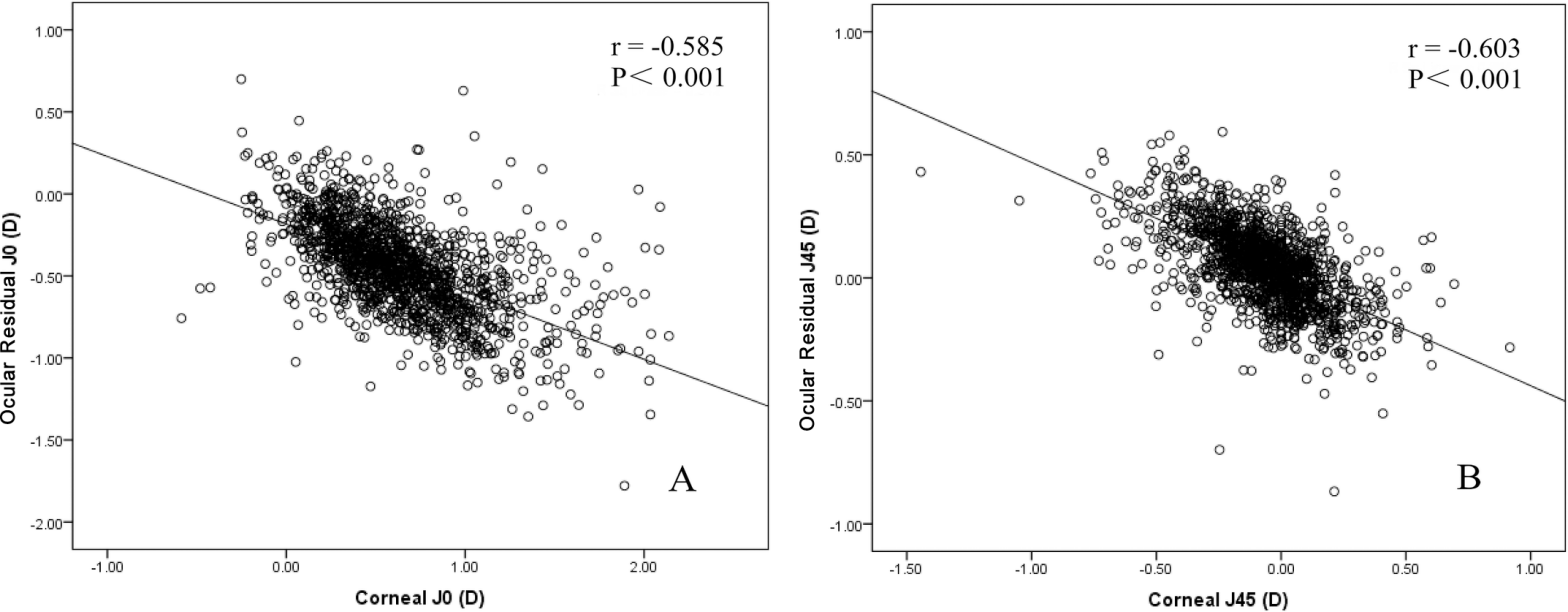
**Correlation between CA and ORA in (A) J_0_ and (B) J_45_.** There was a statistically significant correlation between these two sources of astigmatism for both vectors. J_0_ = Jackson cross-cylinder power at axis 0° and 90°; J45 = Jackson cross-cylinder power at axis 45° and 135°.

Fig 2 shows the correlation between ORA and CA in J_0_ and J_45_. For both vectors, there was a significant correlation between these two sources of astigmatism (r = -0.585, *p* < 0.001 for ORA; r = -0.603, *p* < 0.001 for CA).

### Factors affecting the astigmatism characteristics

Demographic data, including age, gender, and spherical refractive error, were further investigated to determine their potential roles in astigmatisms (Table 7 and Table 8). It was found that subjects presenting with a larger ORA than CA were significantly more myopic than those presenting with a smaller ORA than CA. (-3.14 D vs -2.40 D, *p* < 0.001, Table 7). Nevertheless, no significant difference was found for the age and the gender in respect to the relative level of ORA and CA. In regards to the axis relationship between CA and ORA, none of the demographic data was found to be significant.

**Table 7:** Comparison of demographic factors in respect to the relative level of ORA and CA.

factors	ORA > CA (n = 1094)	ORA ≤ CA (n = 372)	P
Age, median(min,max)^a^	16(15,18)	17(15,18)	0.714
Gender,Male,n(%)^b^	455(41.6)	163(43.8)	0.452
sph, median(min,max)^a^	-3.14(-10.77,5.9)	-2.40(-10.28,3.82)	<0.001

Sph, Spherical refractive error

a. Nonparametric test: Mann-Whitney test.

b. Chi-Square test.

**Table 8:** Comparison of demographic factors in respect to the axis relationship between CA and ORA.

factors	on-axis (n = 10)	opposite-axis (n = 1043)	oblique-axis (n = 413)	P
Age, median(min,max)^a^	16(15,18)	16(15,18)	17(15,18)	0.109
Gender,Male,n(%)^b^	3(30.0)	436(41.8)	179(43.3)	0.638
Sph, median(min,max)^a^	-3.01(-9.26,0.04)	-2.91(-10.77,5.90)	-2.95(-10.66,5.30)	0.837

Sph, Spherical refractive error

a. Nonparametric test: Mann-Whitney test.

b. b. Chi-Square test.

## Discussion

In the present study, we examined the percentage of ORA and CA cases in 1,466 high school students. It was found that ORA was very common in this population, and it usually demonstrated a different axis from that of CA, indicating a compensation relationship between these two major sources of astigmatism of the eyeball.

Overall, the percentage of TA in our samples, given a definition of ≥ 1.00 D, was found to be similar among that measured in adolescents in Hong Kong (21.1%) [26], Taiwan (18.4%) [27], and Singapore (19.2%) [28]. Likewise, in agreement with previous studies, WTR predominated in both CA and TA [29,30], while ATR was the most common type for ORA [31].

In practice, TA and CA can be directly measured by refraction and keratometry, respectively. However, ORA can only be determined indirectly. Around one century ago, Emile Javal [32,33] found an approximately linear relationship between TA and CA and established an estimation formula that was later known as “Javal's rule”: TA = p*CA + k, where p is 1.25 D and k is 0.50 D against-the-rule. After that, Grosvenor and coworkers modified the regression formula according to different circumstances: TA = 0.76 CA—0.40 D for myopic children; TA = 0.84 CA—0.32 D for patients at a university clinic; and TA = 0.87 CA—0.43 D for patients in an optometry practice [32–34].

In recent years, new methods have been introduced to measure ORA, such as by comparing the difference in the cylindrical power between TA and CA through vector analysis [12,35–37] or by comparing the difference in the wavefront between the whole eyeball and the cornea conducted through wavefront analysis. For the latter approach, a sophisticated instrument is usually required, like the KR-1W used in this study. To the best of our knowledge, there is only one other study that has used this approach to investigate ORA, in which patients with senile cataracts were observed [6]. In that study, an ORA of ≥ 1.00 D was detected in 45.5% of all eyes, a similar prevalence to that found in the current study (45.33%). However, there was a significant difference in the distribution of the axis differences between CA and ORA in these two populations. Specifically, the on-axis difference was seen in 10.0%, oblique-axis in 69.4%, and opposite-axis in 20.6% of patients with cataracts, whereas the proportion of each type of axis difference was 0.68%, 28.17%, and 71.16%, respectively, in the high school students in our study. The different distribution pattern between the two studies might reflect the fact that the axis of CA shifts from WTR to ATR with increased age, whereas the axis of ORA becomes WTR from ATR with age [6].

The most interesting finding of this study was that ORA significantly neutralized CA, resulting in a relatively lower amount of TA. Using the parameter of CF, 89.63% of all eyes in the current study demonstrated a smaller TA compared with the CA, indicating that CA can be counterbalanced by ORA. In support of this, it was also observed that opposite-axis was the major type of axis difference between CA and ORA (58.36% for CA and ORA ≤ 1.00 D; 90.96% for CA and ORA ≥ 1.00 D). Additionally, it seems that the compensation relationship between CA and ORA is dependent on the amount, because there was a significant correlation between both vector analyses.

The findings of this study may have significant implications for clinical practice. In fact, uncorrected astigmatism may lead to severe visual discomfort, such as dyslexia and headaches [38]. In addition, reading speed can be influenced by a decrease of up to 24% when an astigmatism is present [39]. Furthermore, driving safety can be significantly improved by correcting low to moderate astigmatisms [40]. Rigid gas permeable (RGP) lenses can be applied to correct corneal astigmatism, but this may not always lead to optimal patient satisfaction. For instance, if the axis of the ORA and CA is opposite, the amount of residual astigmatism will increase after treatment due to the disappearance of the neutralizing effect of the crystalline lens on the CA. Taking this study as an example, 35.68% (523/1,466) of the high school students would have been potential candidates for toric RGP lenses, or soft contact lens, if both CA and ORA ≥ 1.00 D were considered as an indication. It is therefore worthwhile to evaluate the relationship between CA and ORA, with RGP being necessary in cases with a greater amount of ORA.

As mentioned earlier, ORA can only be determined indirectly, either by vector analysis or by the application of a sophisticated instrument such as the KR-1W. Otherwise, practitioners will need a different way to predict the relationship between CA and ORA in daily practice. Based on our findings, we propose the following to help address this issue. First of all, ORA was observed to ascend with the increase of CA in both vectors. Second, the majority of subjects (1,094 out of 1,466) had greater level of ORA than CA. Therefore, for subjects with a substantial amount of CA, they are likely to also have a significant level of ORA that needs a toric contact lens. In addition, we found that subjects with a more negative spherical refractive error, with -3.14 D as the median in the current study, were more likely to have a greater level of ORA than CA. This would help to further differentiate the cases in which toric contact lenses would be recommended.

The results of the present study should be considered in the light of its limitations. One potential limitation might be a lack of a validation of the representation of the subjects in the study, because the subjects were not randomly sampled from the whole population in the city. Nevertheless, as stated previously, the hospital is the exclusive medical unit in which the examinations for the entire city of Anyang are conducted. Additionally, the relatively large number of participants enrolled has lent credence to the representation of the data reported. Another potential limitation might be the lack of a cycloplegic procedure for the KR-1W measurement. Although cycloplegia provides more repeatable inter-visit results, this is mostly applicable for spherical power, and has only a subtle effect on cylindrical power [41–43]. However, the design of the study was not intended to compare values between visits, but rather to decompose the TA for each reading. In support of these findings, the validation study that was conducted prior to the large-scale population study also showed that the astigmatism measurements did not differ significantly after cycloplegia using our measurement conditions.

In conclusion, ORA commonly exists in high school students and usually demonstrates a compensation relationship with CA. As a result, there is a concern that ORA might be exposed if only CA is corrected by spherical contact lenses. For cases with a great amount of ORA, a contact lens with a toric design should be considered in order to increase patient satisfaction.

## Supporting information

S1 Dataset The raw data of all participants. (XLS)
